# Multi-Criteria Decision-Making Approach to Material Selection for Abandonment of High-Pressure High-Temperature (HPHT) Wells Exposed to Harsh Reservoir Fluids

**DOI:** 10.3390/polym17101329

**Published:** 2025-05-13

**Authors:** Augustine Okechukwu Chukwuemeka, Gbenga Oluyemi, Auwalu I. Mohammed, Suhail Attar, James Njuguna

**Affiliations:** 1 School of Computing Engineering and Technology, Robert Gordon University, Garthdee Road, Aberdeen AB10 7GJ, UK; a.chukwuemeka1@rgu.ac.uk (A.O.C.); g.f.oluyemi@rgu.ac.uk (G.O.); s.attar@rgu.ac.uk (S.A.); 2 Technip FMC, Aberdeen AB32 6TQ, UK; a.mohammed12@rgu.ac.uk; 3 National Subsea Centre, 3 International Ave., Dyce, Aberdeen AB21 0BH, UK

**Keywords:** plug and abandonment materials, materials selection, multi-criteria decision making, well integrity, polymer resins, decommissioning, well abandonment

## Abstract

Portland cement is the primary barrier material for well abandonment. However, the limitations of cement, especially under harsh downhole conditions, are necessitating research into alternative barrier materials. While several alternatives have been proposed, the screening process leading to their selection is scarcely discussed in the literature, resulting in the non-repeatability of the selection process. This study develops a dynamic multi-criteria decision-making technique for assessing the material options for the abandonment of high-pressure high-temperature (HPHT) wells with exposure to harsh reservoir fluids. The material screening process is performed in ANSYS Granta and a combined technique for order of preference by similarity to ideal solution (TOPSIS) and analytical hierarchy process (AHP) approach is used for ranking the shortlisted material alternatives based on seven material properties proven in the literature to be critical to the long-term integrity of well barrier materials. Nine alternative materials are ranked against Portland cement and high alumina cement. The results show that the top-ranking materials are from the phenol formaldehyde and polyamide–imide groups. Of these, the primary production CO_2_ of the polyamide–imide is, on average, about 25 times higher than the primary production CO_2_ of the phenol formaldehyde material. A sensitivity analysis of the methodology confirms that the criteria with the highest initial weights are the most impactful in terms of the final rank. The material property values also have an impact on the extent to which variations in their weights affect the hierarchical position of the materials in the TOPSIS-AHP analysis. Despite their higher cost per unit volume, the alternative materials consistently outperformed cement—even when average price was weighted more heavily than the most influential mechanical property.

## 1. Introduction

Plug and abandonment (P&A) is the final stage in the life cycle of oil and gas wells. It is a process aimed at restoring the integrity of the caprock with the help of barrier materials set across sections of caprock zones in the wellbore to prevent fluid migration between incompatible zones of flow potential and into the environment. This process constitutes about 48% of the total decommissioning expenditure forecast for oil and gas assets [1].

To achieve zonal isolation, the oil and gas industry has relied primarily on Portland cement as a barrier material [2]. This product is manufactured from a mixture of limestone, clay and additives through a grinding and clinkering process, with a main chemical composition of dicalcium silicates, tricalcium silicates, tricalcium aluminates and tetracalcium aluminoferrite in about 51.2% of C_3_S, 27.0% of C_2_S, 2.3% of C_3_A and 14.4 of C_4_AF [3]. The industry’s reliance on Portland cement is mainly a result of its robust experience with Portland cement in well construction and in producing wells but also, to a large extent, because well abandonment regulations have been developed based on Portland cement.

Regardless of the wide application of Portland cement and its various derivatives, challenges associated with cement as a well barrier, especially under harsh downhole conditions (high to ultra-high temperatures, high pressures and exposure to naturally occurring or injected corrosive fluids), have been reported in several studies [4,5,6]. This is evidenced by the sustained casing pressures and hydrocarbon leakages in producing and abandoning wells [7,8]. These challenges are key drivers of studies into alternative barrier materials such as thermosets, thermoplastics, certain groups of metallic alloys and geopolymers. While these material options are listed in P&A guidelines and regulations, a comparative analysis of their performance is often not provided.

To understand the key strengths and weaknesses of each barrier material and the impact of harsh downhole conditions on the long-term integrity of barrier materials, [9] conducted a comprehensive review of the literature, including experimental studies on each material type and case histories of their application in P&A projects in mature basins. The study highlights the volumetric shrinkage of Portland cement and its interaction with reservoir fluids as major contributors to the fluid leakages and sustained casing pressures in oil and gas wells. These findings agree with the conclusions of [10,11].

While most of the reviewed works focused on material behavior, Chukwuemeka et al. [9] pointed out the potential contribution of barrier placement methods and placement depths to barrier failures in plugged and abandoned wells.

It is also established in the literature that the cement–casing and cement–formation interfaces present the highest leakage probabilities in abandoned wells [5]. In addition, the exposure of well cement to forces arising from hydraulic fracturing in some wells could lead to the development of fractures and micro-channels for hydrocarbon leakage [12]. In addition to this, mobile formations such as salts and creeping formations are identified as a possible source of cement integrity loss in oil wells [13].

Hitherto, the industry relied significantly on experience and case histories when choosing barrier materials for use in P&A. However, with 871 wells in the UK classified as suspended and inactive for more than the regulatory-approved 2-year period [14] and more wells nearing their cessation of production globally, the development of a comprehensive barrier materials selection strategy for long-term post-abandonment integrity has become a necessity, especially for HPHT wells with exposure to harsh corrosive fluids. Alternative approaches include the application of multi-criteria decision-making (MCDM) techniques and the adoption of artificial intelligence in material selection for P&A. The application of multi-criteria/attribute decision-making (MCDM/MADM) tools for the selection of materials for oil and gas applications has been studied in the literature. For example, using this approach, Mohammed et al. [15] found viable alternative materials that outperform the API-recommended P110 casing grade for shale wells. Through an ANSYS-based simulation study, the authors found that BS S145 has the most outstanding performance of the viable alternatives within the operational boundaries of the given scenario. Additionally, Lavasani et al. [16] developed a fuzzy MADM environment for the selection of plug materials for offshore wells during drilling and production operations in situations that require the synthesis of quantitative and qualitative parameters arising from the diverse opinions of experts. This research develops an MCDM approach suitable for the selection of well abandonment materials.

While several materials have been investigated as alternative barrier materials for well P&A, discussions on their application focus mostly on property characterization/improvement and performance evaluation, with little to no details about the selection process that led to the choice of material. In the absence of a repeatable materials selection process for well abandonment barrier materials, there is room for the exclusion of materials that could potentially outperform existing options. This could also result in the adoption of expensive materials for the abandonment of wells whose conditions permit the use of more cost-effective material options. A robust and repeatable approach that enables comparative studies, reservoir-condition-based materials screening and hierarchical ranking of P&A barrier materials is therefore necessary to ensure that well abandonment materials possess the properties required to maintain long-term integrity under the prevailing conditions and the predictable conditions that may arise in wells, including CO_2_ storage.

In HPHT conditions, the accelerated degradation and debonding of Portland cement due to thermal stress created by the temperature conditions has been identified as a cause of well integrity issues, especially in wells with exposure to harsh/corrosive reservoir fluids [17,18,19]. In the event of material failure or improper placement, remedial well abandonment operations will be required. This could involve a drill out and re-abandonment or a remedial squeeze operation. In a low-cost option for drilling out hardened Portland cement and a metallic plug using coil tubing, Bogianto et al. [20] reported about USD 500,000 being spent on approximately 2200 ft (670.6 m) of hard cement and metal plug. This operation was completed in 24 days. During well abandonment, a single cement plug could reach up to 500 ft to achieve 100 ft of good cement depending on the operator.

The material drill-out cost is only a fraction of the remedial cost for the failure of well abandonment materials. The cost of the redeployment of barrier materials, the health and environmental implications of hydrocarbon leakage and the social/reputational damage for companies are other factors. The remediation cost could vary significantly depending on the location of the well, the barrier materials and the barrier placement depth [21]. Achieving long-term integrity in abandoned wells with exposure to harsh conditions is therefore critical and requires careful consideration of the materials through a multi-criteria decision-making approach.

In this study, materials are screened using the ANSYS Granta materials selector and, subsequently, a combined TOPSIS-AHP approach is used for the ranking of the shortlisted candidate materials as potential permanent barriers for HPHT wells with exposure to aggressive substances, including brine and weak acids. The long-term behavior of these materials in the presence of residual hydrocarbons, represented by organic solvents, and in the presence of strong acids that may present a special challenge in certain wells is also considered in this study. The material properties used in this study include the compressive strength, fracture toughness, thermal distortion resistance, material price per unit volume, shear and bulk modulus and Poisson’s ratio.

## 2. Material Selection for Engineering Design—A Brief Overview

Material selection is the fourth step in a nine-stage process for developing engineering products [22]. In applications such as P&A, where the selected materials are expected to retain their integrity eternally, recycling is not considered a major factor in the design process.

Materials for engineering designs can be selected from over 60,000 useful metallic alloys and nearly as many non-metallic engineering materials. These materials are generally subdivided into six main groups, which include metals, ceramics (technical and non-technical), polymers, composites, semiconductors, and biomaterials [23].

As a result of the significant differences in both the physical and chemical properties existing between and within material groups, the design engineer must define a set of criteria for the material selection process. This can range from a few to several criteria depending on the design objectives. In certain instances, selecting the best-performing material could result in striking a balance between conflicting criteria [24], and poorly selected materials have been identified as a leading cause of engineering components’ failure [25,26]. Other causes of failure include deficiencies in the design, manufacturing defects, overloading of components, poor maintenance and any combination of these [22].

The material selection stage in engineering design aims to deliver optimum performance in both the short and long term at the best possible price through the following steps: (i) translate the design requirements into specifications for materials and processes; (ii) screen out materials that do not meet these defined requirements; (iii) develop a hierarchical ranking of the remaining materials to determine the material with potential for the best performance, and (iv) provide links to additional resources concerning the selected materials to enable a comparative analysis of information such as their relative availabilities, manufacturers, eco-friendliness and environmental impact assessments, etc. [27]. The environmental impact of the extraction and processing of materials has become increasingly important in the face of the global warming challenge and net-zero goals of nations and companies.

The development of material selection strategies over the years is well documented in the literature [28,29,30,31,32]. These studies evolved from structured material data sheets to computer-aided materials and process selection applications, which provide additional information about materials such as their use case histories, manufacturers, and availability, amongst others.

**Figure 1:**
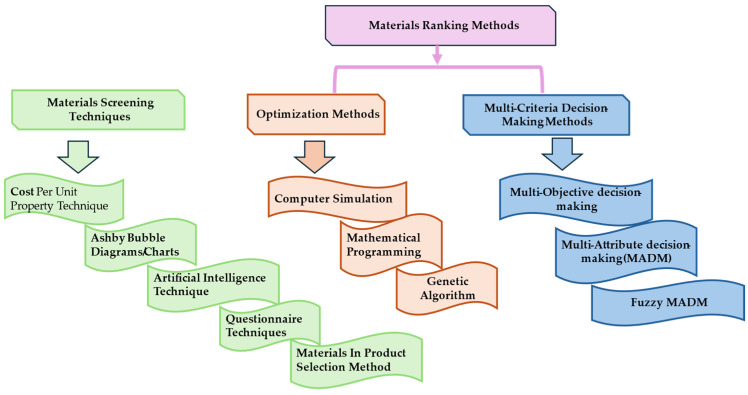
A summary of the material screening and ranking techniques and methods. Adapted from [33].

Arriving at the best material for a specific engineering application involves a material screening and ranking process [33,34]. The screening tools shortlist candidate materials based on the design input parameters. The properties of these materials are then fed into the ranking tools to generate a hierarchy of materials for a specified application. The current state of screening and ranking techniques is summarized in Figure 1 in their broad categories and has been discussed in several studies. These include the analytic hierarchy process (AHP), Hambali et al. [35], simple additive weighting (SAW), Lubis et al. [36], elimination and choice expressing the reality (ELECTRE), Hassan et al., Exconde et al. [37,38], analytic network process (ANP), ANSYS Granta selector, Ferro and Bonollo [39], preference ranking organization method for enrichment of evaluations (PROMETHEE), Maity and Chakraborty [40], complex proportional assessment (COPRAS), Chatterjee et al. [41], technique for order performance by similarity to ideal solution (TOPSIS), Huang et al., Rahim et al. [42,43], VlseKriterijumska Optimizacija I Kompromisno Resenje (VIKOR) [44] and the Ashby method [29].

The above techniques for material selection have been critically reviewed by [23], who classified them as either screening or ranking methods, highlighting the advantages, disadvantages, and areas of popular application of each method. In addition to these, other techniques that are standalone derivatives or combinations of the above methods have also been applied for material ranking and selection. For instance, various adaptations of the above methods for materials selection in fuzzy scenarios have been reported in the literature. In their study, Mayyas et al. [45] applied fuzzy TOPSIS in an eco-material selection exercise. Fuzzy VIKOR and fuzzy PROMETHEE have also been applied by [46,47] in various material selection studies.

The ANSYS Granta software is one such tool built on the principles of the Ashby method, which utilizes the material property ratios and performance indices for assessing alternative materials under defined conditions [48]. In ANSYS Granta, the property ratios and performance indices are transformed into bubble diagrams for easy visualization during the selection process. It combines a structured database of materials, material properties and applicable processing methods with an information management system that has links to external sources of the manufacturers and the availability of these materials. The Process–Property profiles datasets allow users to explore the effect of processing on the material properties [27]. This combination enables flexibility for trade-offs, modifications, and combinations for hybrid designs. In this tool, the best material alternatives are the ones with the highest performance indices. To arrive at this shortlist of alternatives, the software accepts design inputs and provides a platform for users to define the limit values for material properties.

A study by [34] used the Ashby charts for the selection of plant-fiber-reinforced plastics for product design and improvement as alternatives to glass fiber composites and demonstrated the tool’s robust capabilities in assessing the mechanical properties of materials, the processing methods applicable to each material and how each material’s eco impact compares with those of other materials. Readers may refer to the following literature for additional use cases of the ANSYS Granta and Ashby plots for material selection [49,50,51].

The shortlisted alternatives are ranked using either analytical techniques, further study through finite element analysis or experiments to provide additional assurance against product failures. Typical examples of such analytical MCDM techniques are the technique for order preference by similarity to ideal solution (TOPSIS) and analytical hierarchy process (AHP). As an MCDM technique, TOPSIS, which was developed by [52] for the optimal selection of the best alternative solution given a finite number of criteria, provides a simultaneous ranking of the alternatives in order of their relative distances from the positive ideal and negative ideal solutions. These distances are calculated using mathematical equations, numerical values of the considered criteria and weights assigned to the decision criteria based on expert judgement. On the other hand, AHP, which was introduced by [52,53], provides a simple structured solution to decision-making challenges. In this process, a pairwise comparison of the alternatives is developed and checked for accuracy through a consistency ratio.

## 3. Methodology

In this work, the Ashby method is used for the material screening in the ANSYS Granta Edupack 2024R1 software, while a combined TOPSIS-AHP approach has been adopted for the ranking of the shortlisted materials as barrier materials for the plug and abandonment of HPHT oil and gas wells. A complete TOPSIS ranking process is conducted on the shortlisted materials, followed by the TOPSIS-AHP ranking for the validation and comparison of the results. The choice of MCDM tools is informed by their availability, robustness, scope of information provided, simplicity of use and proven record of accuracy in engineering applications. For its part, TOPSIS is a 6-stage process and is easy to use without requirements for high-level expertise beyond the phase of criteria definition and the assignment of weights to the selection criteria. This is suitable for fast-paced environments such as the oil and gas industry and a major reason for its choice as a tool for this study. However, TOPSIS, as a standalone MCDM tool, is prone to errors arising from biasness in the weights assigned to the ranking criteria.

An integrated TOPSIS-AHP approach is, therefore, adopted for the final ranking of the materials in this research to avoid the possibility of errors that could potentially arise from biasness in the assignment of the weights to the selection criteria in TOPSIS. This is achieved with the help of AHP’s consistency ratios derived from its pairwise comparison process. In addition to this, a sensitivity study is conducted to determine the impact of variations in the criteria weights on the final ranking of the shortlisted materials.

**Figure 2:**
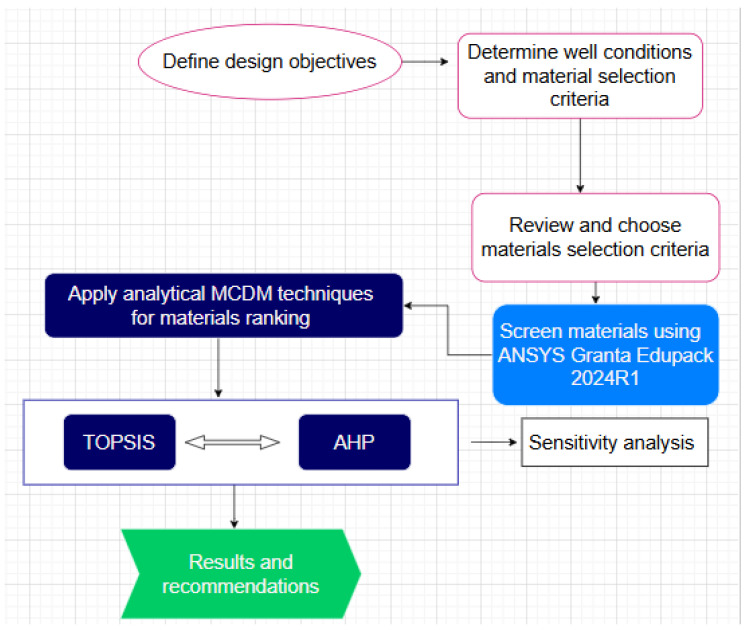
A summary of the methodology undertaken in this study.

Figure 2 below is a summary of the key stages that make up the adopted methodology for this work aimed at selecting reservoir barrier materials for permanent abandonment of HPHT oil and gas wells.

The following sections will discuss the process of applying these techniques in greater detail.

### 3.1. Ashby Method in ANSYS Granta Materials Selector

The material screening in this research is conducted on the level-3 aerospace materials database in the ANSYS Granta Edupack 2024R1 software for the initial screening of over 40,000 materials to shortlist candidate reservoir barrier materials for the P&A of HPHT wells. This group has been selected because of its wider database of materials and inclusion of high-performance materials for extreme-temperature applications. The screening process involves the plotting of two-dimensional graphs of the performance criteria and indices to generate an array of materials in the form of bubble diagrams also known as Ashby plots. To generate these diagrams, a combination of defined material properties, limit conditions and performance indices are synthesized by the ANSYS Granta software.

Material properties such as the compressive strength, fracture toughness, etc., which are quantified and have numerical values, have been plotted on the xy coordinates of the bubble diagrams to provide a means of visual comparison of the materials.

Additional performance requirements that represent the expected operating conditions of the selected barrier material are defined in the software using its limit function. The typical operating conditions expected in depleted HPHT reservoirs during and post abandonment include a temperature range of 130 to 200 degrees Celsius and pressure values that could potentially recharge to 10,000 to 15,000 psi [54]. Additionally, the presence of a hydrocarbon/brine mixture, weak acids and strong acids occurring naturally or from well interventions for enhanced oil recovery are common in depleted reservoirs. Given that the software does not name specific acids under its limit function, the general classes available in the software have been used for this research and the organic solvents class has been adopted to represent hydrocarbons.

To account for the impact of these fluids and conditions, an excellent performance rating is selected for all the identified reservoir fluid groups except for strong acids, as shown in Table 1.

A material is considered to show excellent performance in each solution under specific conditions if no degradation in material performance is expected after long-term exposure. Limited-use materials are generally not recommended but may be suitable for short-term applications, while acceptable materials may require additional protection for long-term performance under the selected operating conditions. However, materials with limited use and acceptable performance could be improved with the help of additives to meet the design objectives. Further information on this classification of materials based on their longevity in the solutions is provided in Section 4. The wider performance scope selected for strong acid is because the acids occurring in the reservoirs are diluted by the reservoir water. The combination of limiting conditions used in this research to define the expected operating conditions of the selected barrier material and their values are listed in Table 1.

Given the oil and gas industry’s robust experience with pumping technologies for the deployment of barrier materials in wells, the materials processing function in the ANSYS Granta software is utilized to screen out non-injectable materials. The shortlist of material alternatives from ANSYS Granta is then ranked using TOPSIS and the combined TOPSIS-AHP technique.

### 3.2. Methodology for Ranking Materials Using TOPSIS and Combined TOPSIS-AHP

#### 3.2.1. TOPSIS Method

The mathematical procedure for arriving at the materials ranking in TOPSIS is broken down into the following stages:

(a) Decide on and assign weights to individual criteria considered for the material ranking process based on expert opinion.
(b) Calculate the normalized and weighted normalized values for the material selection criteria using the Euclidean vector normalization approach.
(c) Determine the positive and negative ideal material solutions based on the benefit and non-benefit attributes.
(d) Calculate the separation measures for each material from the positive and negative ideal solutions. This is basically their Euclidean distances form these solutions.
(e) Calculate each material’s similarity to the positive ideal solution.
(f) Rank the materials based on their distances/similarities to the positive ideal solution.

The best material for the project based on the chosen set of criteria and assigned weight values is the material closest to the ideal solution. For the general TOPSIS equations, readers may refer to [54,55], from which the equations used in this section are adapted. In order to reduce the possibilities of biasness without the impact of information overload discussed in [56], 9 materials covering the families of polymers arrived at through the screening exercise in the ANSYS Granta software are ranked against Portland cement and high alumina cement (HAC) as alternative barrier materials for the plug and abandonment of HPHT oil and gas wells with exposure to corrosive fluids based on a set of 9 criteria. Given these 11 alternatives and the 9 criteria, letter A is assigned to represent each material alternative and letter y is assigned to represent the positive real number values of each of the 9 selection criteria.

First, weights w1 to w9, which reflect the significance of each criterion to the long-term performance of the material alternatives in an HPHT well are assigned to each criterion y1 … y9, as shown in Table 2. The weight values are derived from the established significance of the selection criteria as published in the literature, industry standards, Oil and Gas UK’s guidelines on the qualification of materials for well abandonment published in 2015 and the properties often tested by developers of alternative barriers. The w values sum up to unity as required in TOPSIS. Criteria y1 to y8 are considered benefit criteria, while y9 (average material cost) is considered a non-benefit criterion for every material alternative.

Put together, the resulting matrix is an 11 × 9 matrix Y of all the *y*1 *… y*9 values for each of the material alternatives from A1 to A11.

(1) $$Y=\begin{array}{ccc} y_{11} & y_{12} & y_{13}\ldots y_{19}\\ y_{21} & y_{22} & \ldots \ldots y_{29}\\ y_{31} & y_{33} & \ldots \ldots y_{29}\\ \vdots & \vdots & \vdots \\ y_{11,1} & y_{11,2} & y_{11,9} \end{array}$$

Given that the units of measurement for these parameters are different, for each random criteria *ymn*, in the matrix Y above, a normalized value, Rmn, is calculated using the following equation:

(2) $$Rmn=\frac{y_{mn}}{\sqrt{\sum_{m=1}^{11}\left(y_{mn}^{2}\right)}}$$

These normalized values are within the range of 0 to 1.

The selected criteria and assigned weights can affect the ranking of materials in TOPSIS.

To factor in the impact of the assigned weights on the final ranking of materials, the normalized *r* values are replaced by the weighted normalized values amn.

Where amn = rmn × wn(3)

Both the a and r values lie within the open interval of $\langle 0,1\rangle .$ A decision matrix A containing these normalized weighted values is generated for further calculations in the TOPSIS analysis.

(4) $$A=\begin{array}{ccc} a_{11} & a_{12} & a_{13}\ldots a_{19}\\ a_{21} & a_{22} & \ldots \ldots a_{29}\\ a_{31} & a_{33} & \ldots \ldots a_{39}\\ \vdots & \vdots & \vdots \\ a_{11,1} & a_{11,2} & a_{11,9} \end{array}$$

For further data processing and calculations, the coordinates of the positive ideal solution A* = $\left(a_{1,}^{*}a_{2}^{*},a_{3}^{*}\ldots a_{9}^{*}\right)$ are calculated using the following formulas:

$$\underset{m}{\max}a_{mn}\;for\;n=1,2,\ldots ,8$$

$$\underset{m}{\min}a_{mn}\;for\;n=9$$

For the negative ideal solution Ao = $\left(a_{1,}^{o}a_{2}^{o},a_{3}^{o}\ldots a_{9}^{o}\right)$, the coordinates $a_{n}^{o}$ are calculated using the following formulas:

$$\underset{m}{\min}a_{mn}for\;n=1,2,\ldots ,8$$

$$\underset{m}{\max}a_{mn}\;for\;n=9$$

The values of the distances from Am to A* and from Am to A0 are represented by d* and do, respectively, and calculated using the following general formulas for the 11 shortlisted materials:

(5) $$d_{m}^{*}=\sqrt{\sum_{n=1}^{9}{\left(a_{mn}-a_{n}^{*}\right)}^{2}}$$

(6) $$d_{m}^{o}=\sqrt{\sum_{n=1}^{9}{\left(a_{mn}-a_{n}^{o}\right)}^{2}}$$

The values of these distances are utilized to calculate the relative distances, $D_{m}^{*}$, of points Am from the respective points A* and Ao using the equation below:

(7) $$D_{m}^{*}=\frac{d_{m}^{o}}{d_{m}^{*}+d_{m}^{o}}=\frac{d\left(A_{m},A^{o}\right)}{d\left(A_{m},A^{*}\right)+d\left(A_{m},A^{o}\right)}$$

The material A, with the maximum value of D, is accepted as the best material choice, while the material with the corresponding least value is the worst choice in the group of materials.

TOPSIS is a simple-to-use tool for material selection, as shown by the number of stages involved in the calculations and their simplicity, which has gained popularity amongst researchers and industry professionals. However, an inconsistency in judgement can remain undetected in TOPSIS and lead to consequent errors in the ranking of materials. A likely source of this error is biasness in the opinions that form the basis for the assignment of weights to the material properties in TOPSIS.

Another MCDM technique called AHP, the analytical hierarchy process, is used in combination with TOPSIS in this work to check for and eliminate the possibility of this error.

#### 3.2.2. TOPSIS-AHP Method

The AHP is a popular multi-criteria decision-making technique structured to analyze the subjectivity of decision-makers through a calculated consistency ratio [67]. The AHP is based on pairwise comparison using the fundamental scale of values shown in Table 3 below. In addition, a random consistency index, RI, based on the number of criteria being compared plays a crucial role in the AHP technique. Combining this technique with TOPSIS for the estimation of the importance of the ranking criteria reduces the chances of an undetected error and improves the reliability of the final ranking [68]. This combined technique has been applied by different authors for solving engineering problems [69,70,71]. In the first example, ref. [69] evaluated and ranked nontraditional machining processes using the TOPSIS-AHP approach and proved it to be more efficient than the standalone TOPSIS or AHP. This combined approach was also applied by [70] to optimize and balance the mechanical properties of glass-fiber--reinforced epoxy composites filled with fly ash. It was also applied by [71] to evaluate cobalt-bonded tungsten carbides. The findings of these studies highlight the effectiveness of this approach in solving engineering problems.

In this work, eleven material options are ranked based on seven key criteria (average compressive strength (CS), fracture toughness (FT), thermal distortion resistance, bulk modulus, shear modulus, Poisson’s ratio, and average price). The values of these criteria are extracted from the ANSYS Granta materials database and used for the pairwise comparison of the selection criteria in accordance with Table 3 and Table 4. In addition, a pairwise comparison of the above selected criteria is conducted to determine the priority vector of each criterion. Some of the initial criteria used in TOPSIS with negligible variances across materials have been considered insignificant in this AHP. Given the closeness of the values, these parameters will have an insignificant impact on the pairwise comparison and material ranking in the AHP.

The AHP is based on the pairwise comparison matrix P = || pij || (i, j = 1, 2, …, m). All the evaluation criteria Ri and Rj (i, j = 1, 2, …, m) are compared against themselves, where m is the number of criteria in this study. In this research, m = 7 and the number of alternatives is 11. The elements of this matrix are representative of the relationship between the unknown criteria weights.

The process leading to the final material ranking is divided into the following:

- Define the problem.
- Structure the hierarchy from the definition of objectives through the determination of ranking criteria and composition of a list of alternatives.
- Construct a set of pairwise comparison matrices, size n x n, for each of the alternatives using the relative scale in Table 3.
- Reciprocals are automatically assigned in each pairwise comparison.
- Hierarchical synthesis is then used to weight the eigenvectors by the weights of the criteria. The sum is taken over all the weighted eigenvector entries corresponding to those in the next level of the hierarchy.
- After all the pairwise comparisons, the consistency is determined by calculating the consistency index CI using the formular CI = (*ƛ*max − n)/(n − 1), where n is the matrix size.
- The consistency ratio is acceptable if it does not exceed 0.10. If it is more, the judgment matrix is inconsistent.

In this work, the calculated priority vectors for the selection criteria are plugged into the TOPSIS analysis as replacements for the assigned weights after confirming the consistency ratio of the pairwise comparison is less than 0.1. The results of the above combinations of multi-criteria decision-making tools for screening and ranking materials for the plug and abandonment of HPHT oil and gas wells in a hybrid barrier system are discussed in the following sections.

## 4. Results and Discussion

**Figure 3:**
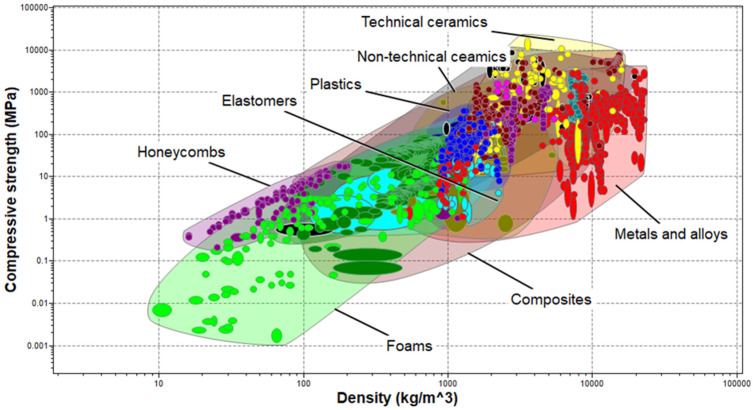
Bubble diagram of material options. Materials from the technical ceramics group occupy the upper band of compressive strength, while materials from the metals and alloys group occupy the extremes of the density axis.

A combination of Ashby plots based on the predetermined relevant material properties and limit performance criteria inputs, as in Table 1, applied to materials in the level-3 aerospace database of the Ansys Granta material selector were generated in the screening process. The plot shown in Figure 3 compares the compressive strength of the materials against their density. Material groups such as metals and alloys, technical ceramics, non-technical ceramics, etc., are identified within specific envelopes. The array of materials in Figure 3 shows that materials with close density values could have significantly different compressive strengths. While the material density could vary at the phase transition, its value for the liquid phase of barrier materials has a direct impact on the hydrostatic pressure exerted by the material during pumping. Sufficient hydrostatic is needed for primary well control. However, extreme overbalance could lead to reservoir fracture. On the other hand, compressive strength is linked to the permeability of materials in a study by [58], which investigated the impact of silica fumes on natural clay liners. The compressive strength of natural clay was found to increase, while its permeability and swelling pressure decreased, on the addition of silica fumes at greater than or equal to 25 percent. In a study of five barrier materials options, including an industrial class of expansive cement, a non-cement pozzolanic slurry, a rock-based geopolymer and an organic thermosetting resin, ref. [72] found that the uniaxial compressive strength of the thermosetting polymer after seven days of curing was 130 MPa, a value three times greater than the uniaxial compressive strength of class-G Portland cement cured under same conditions for the same period. A drop in the value of the compressive strength of the organic thermosetting polymer to 80 MPa was also observed after 28 days. These reported changes require further investigation to better understand the long-term performance of barrier materials for P&A applications.

In addition to the density and compressive strength, the fracture toughness of materials, which defines their ability to resist the initiation and propagation of cracks through the material matrix, contributes to the load-bearing capacity and integrity retention of well abandonment materials.

**Figure 4:**
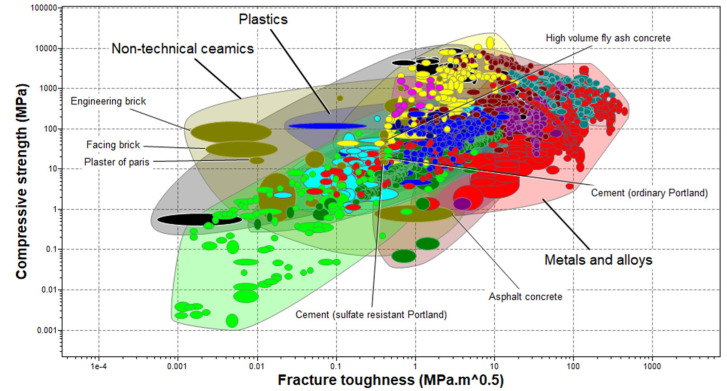
Array of level-3 aerospace materials—A comparison of the compressive strength to fracture toughness of the materials.

To investigate the ability of materials to resist fracture in the presence of compressive loads, the compressive strength of the materials in the level-3 aerospace materials group is plotted against their respective fracture toughness. As seen in Figure 4, the relationship between the compressive strength and the fracture toughness is not linear. This is significantly evident in the non-technical ceramics group, where engineering brick has a higher compressive strength than asphalt concrete but a lower fracture toughness. This is corroborated in [73], which found a non-linear relationship between the compressive strength and the fracture toughness of Al_2_O_3_ whisker-reinforced alumina-toughened zirconia (ATZ) and zirconia-toughened alumina (ZTA) nanocomposites. It is therefore important to strike a balance between the compressive strength and the fracture toughness of materials in the selection of a suitable P&A barrier materials for HPHT oil and gas wells.

Furthermore, the non-numeric or qualitative properties of the materials in Table 1 were applied to the selection process to initiate a material screening process in the ANSYS Granta software. On applying the temperature, compressive strength boundaries and durability in fluid conditions, materials such as ordinary Portland cement and sulfate-resistant Portland cement grayed out in the compressive strength–fracture toughness bubble diagram. An explanation of the classification of materials based on their durability in a given medium is found in Table 5.

**Figure 5:**
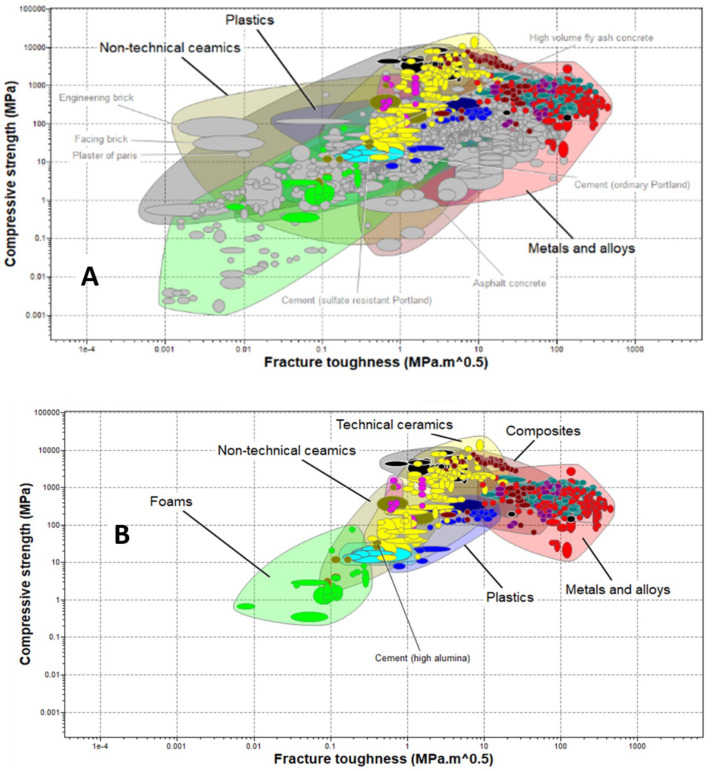
Bubble diagrams of level-3 aerospace materials on a compressive strength–fracture toughness plot after applying the limit conditions indicated in Table 1. (**A**) shows all the materials, including failed records, while in (**B**), failed records are screened out.

These grayed-out materials, shown in Figure 5A, do not meet one or a combination of the limit criteria set in this study. A similar approach was adopted in [15] for the selection of high-performance casing materials for application in shale oil and gas wells.

For strong acids, the limiting performance conditions used in this research include excellent, acceptable, and limited use. This expanded performance scope has been selected to reflect the fact that acids occurring in mature reservoirs are often diluted by reservoir water. This is deduced from [65], which studied wells in the Campos Basin and found that water production in late-life wells could reach three times the volume of the produced hydrocarbons. Amosa et al. [66], in a study of wells in the Norwegian continental shelf, also showed that the sulfur content by weight in crude oil and the hydrogen sulfide content in natural gas are in the zone of 0.3 to 0.8 percent and 0.01 to 0.4 percent, respectively. The same study investigated the relationship between the reservoir temperature and hydrogen sulfide in hydrocarbons produced from wells in the continental shelf of Norway. The authors discovered that above 110 °C, the hydrogen sulfide concentration in hydrocarbons from this area increases exponentially. Candidate wells for plug and abandonment are usually at the end of their economic life. At this stage, production from the well is a mixture of higher percentages of produced water and hydrocarbon, which justifies the inclusion of materials with acceptable and limited-use performance ratings in the materials selection criteria for performance in strong acids.

In addition to the above, expanding the limit function to include materials outside the excellent performance zone allows room for the inclusion of materials whose properties could be improved with the help of additives to achieve the desired performance levels in strong acids. The application of additives for the improvement of the chemical resistance of barrier materials against acid attack has been proven in different studies. In a study to examine the impact of graphene oxide, GO, on oil well cement using field emission scanning electron microscopy morphology tests, Chintalapudi and Pannem [74] found that the addition of 0.04% of GO improved the resistance of cement to sulfuric acid through the formation of flower-like crystals. This led to improvements in the microstructure and hydration properties of the tested cement samples after 28 days of exposure. Another study, Srivastava et al. [75], on the impact of magnesium oxide on cement under varied temperature and CO_2_/methane concentrations in brine, found that between 38 and 177 degrees centigrade, the impact of magnesium oxide on the resistance of the tested class-H Portland cement varied with the curing temperature, pressure, and CO_2_ concentration. Higher pressures led to increased solubility in the gas and hence increased the carbonation of the cement in a test period of 14 days. While these are very short aging periods compared to the expectations in well abandonment, the findings of these tests suggest that the careful selection and application of additives to materials with limited and acceptable performance ratings in relation to strong acids could improve their overall performance in abandoned wells with significant exposure to such acids. Such material property improvement studies are, however, beyond the scope of this research.

Furthermore, a compressive strength of 80 MPa was applied as an additional limiting factor in this material selection study. This value reflects the upper extreme of the unconfined compressive strength (UCS) for North Sea shale caprocks. According to [54], the UCS values for shale in the North Sea ranges from 3.5 to 86 MPa. This performance constraint screens out high alumina cement (HAC), which is rated as having a long-term excellent performance in weak acids, brine and strong acids. Several studies in the literature validate the performance of high alumina cement in the presence of acids. This is a major factor driving its extensive application in the construction of sewer pipes and pipe linings where exposure to aggression from sulfate and biogenic sulfuric acid is a common occurrence [76]. Its stability in acids has been attributed to the absence of calcium hydroxide, the presence of more stable calcium aluminate hydrates and the formation of aluminum hydroxide [77,78]. However, a study by [79] in which the performance of ordinary Portland cement, HAC, and geopolymer cementitious material in HCl and H_2_SO_4_ of a pH equal to 3 indicated a rapid mass and compressive strength degradation for HAC over a study period of 24 months. At about 480 days and 360 days, the compressive strength of HAC falls below the compressive strength of the compared Portland cement in HCl and H_2_SO_4_, respectively. These results highlight the need for the inclusion of the specific operating conditions and environment in the material selection process.

In addition to the above selection criteria, the placeability of barrier materials in the well is a major factor in plug and abandonment. Given the level of advancements in the pumping technologies used for oil well cementing, the material universe for this research is at this stage restricted to plastics and non-technical ceramics to improve the chances of selecting materials that could be placed in wells using existing technologies. The remaining materials are further examined in an Ashby plot of the thermal distortion resistance and Poisson’s ratio, which are relevant for the long-term barrier performance and zonal isolation in HPHT wells.

**Figure 6:**
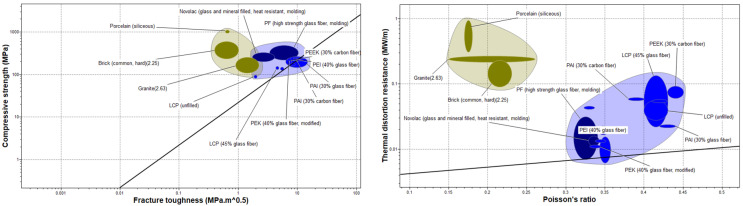
Bubble diagrams demonstrating the use of the performance index for the optimal screening of materials.

Performance index lines whose slopes measure the ratio of compressive strength to fracture toughness of materials and thermal distortion resistance to Poisson’s ratio of materials are plotted on the corresponding bubble diagrams, shown in Figure 6, to optimize the screening process. Moving the index lines on the bubble diagrams screens out those materials that fall below the performance index. The remaining group of materials are suggested as the potential best-performing materials for the abandonment of HPHT wells.

A clear distinction in the properties of ceramics and polymers is observed in this bubble diagram. While the polymers have higher values of Poisson’s ratio, the ceramics possess higher values of thermal distortion resistance. However, according to [13], materials with higher Poisson’s ratio values are preferred for improving the zonal isolation. This is attributed to the impact of Poisson’s ratio on hoop stress, which becomes more tensile as Poisson’s ratio decreases [13]. While these studies were performed on class-G and -H oil well cement, they point to the advantages of a higher Poisson’s ratio in terms of the performance of well barriers. Regardless of their high compressive strengths, the ceramic materials in Figure 6 are either too brittle, in the case of porcelain, or too dense, in the case of bricks. These could pose long-term integrity and deployment challenges in well abandonment. However, they could be used as additives for the improvement of the compressive strength of other barrier materials. For example, Bignozzi and Saccani [80] found that porcelain waste could be used to improve the compressive strength of concrete by up to 41 percent and its tensile and flexural strengths by 41 and 67 percent, respectively. Sprenger [81] also found that the use of porcelain residue in cement limits the alkali silica reaction in concrete.

As a result, the polymers in Figure 6 are selected over the ceramic materials for further analysis using a combined TOPSIS-AHP approach. The output of this process is a hierarchical ranking of the selected materials as potential barriers for the abandonment of HPHT wells. The material property values, as shown in Table 6, used in this research are extracted from the ANSYS Granta material selector. These materials are benchmarked against Portland cement and high alumina cement for a comparison.

### Material Ranking by TOPSIS and TOPSIS-AHP Process

Nine materials shortlisted with the help of the Ashby plots are further studied alongside ordinary Portland cement and HAC using TOPSIS and a combined TOPSIS-AHP multi-criteria decision-making process, as described above.

Nine material properties shown in Table 6 are used in the TOPSIS analytical process for ranking the materials. Numerical values are assigned to non-numerical properties such as stability in brine, stability in weak acids, etc. The choice of numbers from 1 to 4 represents the classification of materials based on their durability in each fluid. In this scenario, 1 represents unacceptable use, while 4 represents excellent use. Given that all the selected materials except Portland cement have excellent performance in water, brine and weak acids, these performances are combined into one by obtaining their arithmetic mean to reduce the number of criteria and simplify the analysis.

The equations in Section 3 are built into an Excel spreadsheet to determine the proximity of each material to the positive ideal solution and their relative position from the negative ideal solution. This distance is then used to determine the hierarchical ranking of the alternatives.

Prior to the calculation of the distances from the ideal solution, the sum of the assigned weights of the criteria was determined. As seen in Table 7, this value equals 1. This is a required compliance criterion in the TOPSIS MCDM analysis.

The best-ranked barrier materials from this TOPSIS ranking are from the PF (phenol formaldehyde) and PAI (polyamide–imide) groups. In comparison to Portland cement and HAC, which are ranked 10th and 5th, the PF (high-strength glass fiber, molding) and PAI (30% carbon fiber) rank 1st and 2nd, respectively, for the design application.

In the combined TOPSIS-AHP approach, the assigned weights in TOPSIS are replaced by the priority vectors calculated as discussed in Section 3. Table 8 presents the result of the pairwise comparison of the selection criteria. Given that the numerical values representing the performance of the shortlisted materials in water, brine, weak acids, and organic solvents are constant and all the shortlisted candidate materials meet an excellent performance criterion in terms of them, these properties are not included in the pairwise comparison carried out in this work. This reduced number of criteria improves the consistency in judgment. The consistency ratio achieved in this study is 0.033, which meets the standard requirement for accuracy in the AHP. A consistency ratio of less than or equal to 0.1 is required for any AHP study [53].

By replacing the assigned weights in the TOPSIS analysis with the priority vectors calculated in Table 8, a new ranking of materials is obtained. In this combined TOPSIS-AHP analysis shown in Table 9, the top-two ranking materials from TOPSIS remain top but flip their order in the hierarchy. PF (high-strength glass fiber, molding), which ranked first in the standalone TOPSIS analysis, now occupies the second position in the combined TOPSIS-AHP, giving up the first position to PAI (30% carbon fiber). Further changes in the ranks are observed for other materials in this study. For instance, HAC, which hitherto ranked fifth in TOPSIS, ranks ninth in the combined TOPSIS-AHP.

**Figure 7:**
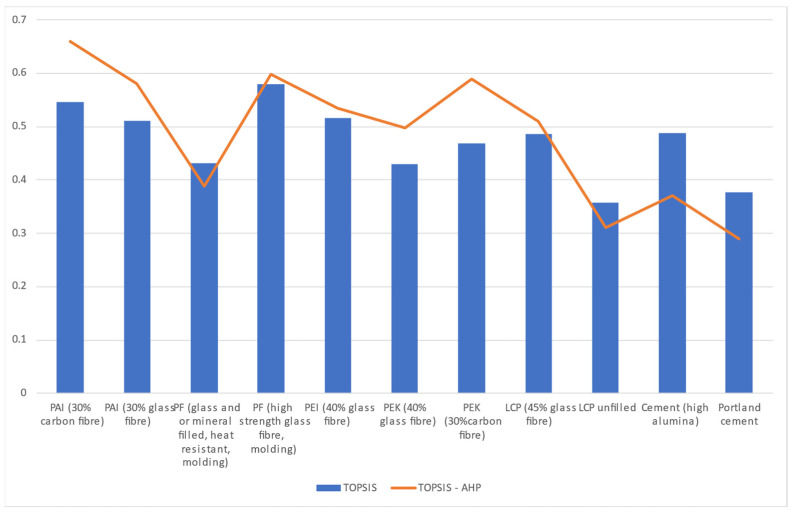
Comparison of materials rankings as permanent plugs in HPHT wells using TOPSIS and combined TOPSIS-AHP. The top 2 and bottom 2 material groups remain unchanged while significant rank changes are observed.

Regardless of these variations, a comparison of the results from the two studies shows that the two best materials and the two worst materials have retained their positions as the best and worst groups, which inspires confidence in the ability of either MCDM method to help the design engineer to identify the best and worse materials for this design. The positional changes in the ranking of materials are represented in Figure 7. Although the Novolac and PF (high-strength glass fiber, molding) materials are from the same broad phenol formaldehyde family, a significant difference in hierarchy is observed between them. This difference is attributable to the sharp variation in the respective values of their compressive strength and fracture toughness. Apart from these, both materials possess similar values for other properties that formed the selection criteria in this research.

Altering the ranked material alternatives through the addition of additives could improve their positions in the ranking order. This is achieved though improvements in material properties achieved with the addition of additives. For instance, the application of surface-modified silica nanoparticles for mechanical property improvement in reinforced epoxy resins has been studied extensively in the literature. For instance, Makeev et al. [82] showed an increase in the modulus and strength of resins following the addition of silica nanoparticles. Similar findings are reported in [83].

**Figure 8:**
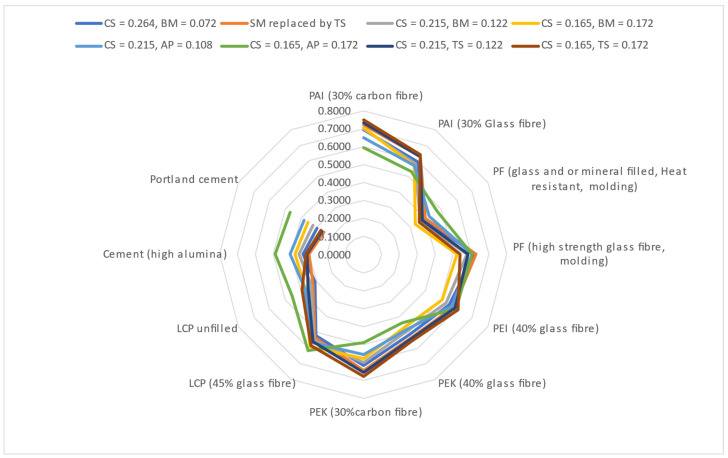
Sensitivity analysis of the TOPSIS-AHP material ranking based on selected material properties. CS—compressive strength, AP—average price, BM—bulk modulus, SM—shear modulus, TS—tensile strength.

To measure the dependence of the material ranking in the TOPSIS-AHP approach on the ranking criteria and assigned weights, a sensitivity analysis is conducted through a controlled variation of the assigned weights and a replacement of the shear modulus by the tensile strength as a ranking criterion. The assigned weights are varied in pairs through an increment of 0.05 in the weight assigned to one selection criterion and a corresponding decrease of 0.05 in the weight value of the second criterion. Figure 8 and Table 10 below show the changes in the ranking order of the materials when the weight values of selected material properties are modified and when the shear modulus of the materials is replaced by their tensile strength. When the shear strength values of the materials are replaced by the corresponding tensile strengths, the D values for all the ranked materials increased except for cement (high alumina) and Portland cement, which decreased instead. The percentage increase ranged from 1.1909 percent for PF (high-strength glass fiber, molding) to a 5.8531 percent increase for LCP unfilled. In comparison, the percentage decrease in D was the highest for Portland cement at 14.7412 percent. While the top-four and worst-three ranked materials remain within these categories, there are internal changes in the ranking within the top-four ranked materials. PEK (30% carbon fiber) moves from third to second position in the ranking order, while PF (high-strength glass fiber, molding) moves from second to fourth position in the ranking order. PAI (30% carbon fiber) retained its number one position in the ranking order in all instances of the sensitivity analysis except in the scenario where the weight factor of the average price became dominant over the compressive strength. In this scenario, PEK (30% carbon fiber) became the best-ranked material, followed by PF (high-strength glass fiber, molding). A decrease in the weight assigned to the compressive strength leads to an increase in the D values for LCP (45% glass fiber), LCP unfilled, cement (high alumina) and Portland cement in all the tested scenarios except when a decrease in the compressive strength weightage is accompanied by a corresponding increase in the weight assigned to the tensile strength of the materials. In this circumstance, the D values of high alumina cement and Portland cement are reduced by 4.28 and 8.77 percent, respectively, from their initial values. On average, an increase of 0.05 (0.122 to 0.172) in the weight of the tensile strength with a corresponding 0.05 (0.215 to 0.165) decrease in the weight of the compressive strength generated a 3.6 increase in the absolute average percentage change in the D values across the ranked materials. However, the same variation of weights between the bulk modulus and the compressive strength results in a 6.3 increase in the absolute average percentage change in the D values of the ranked materials. This indicates that the material property values have an impact on the extent to which variations in their weights affect the hierarchical position of materials in the TOPSIS-AHP analysis.

In all the tested scenarios, a material from the thermoplastic family ranks better than the rest of the alternatives. However, it is important to note that their deployment and curing mechanisms under downhole conditions require additional investigation.

The compressive strength and average price of the materials have the most significant impact on their ranking. This agrees with the findings of [68], namely that the stability of the ranking order when the weightage of the ranking criteria is altered is affected by the initial magnitude of the weights assigned to the materials. In addition to having the highest and lowest initial ranking weights, 0.265 and 0.058, respectively, the compressive strength and average price of the ranked materials also have higher numerical values when compared to the other ranking criteria.

A total order reversal in which an initially worse-ranked material becomes the best-ranked alternative is not observed in any of the scenarios considered.

**Figure 9:**
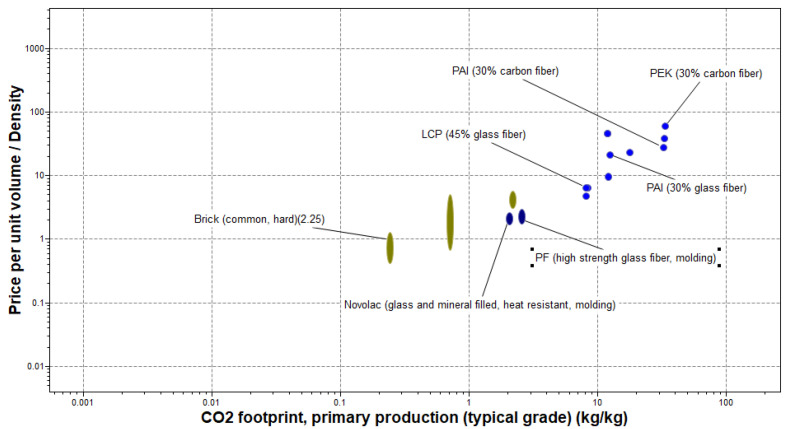
Environmental impact/economic feasibility assessment.

For further understanding of the suitability of the top-two materials for the plug and abandonment of HPHT wells according to this study, bubble plots of the average material cost and their primary production CO_2_ emission are investigated. The CO_2_ emission factor, which was not considered in the earlier stages of this material selection process, investigates the degree of compliance of well abandonment materials with the net-zero targets of oil and gas companies and regulators. Figure 9 shows the difference in price and the production carbon footprint for PF (high-strength glass fiber, molding) and PAI (30% carbon fiber). For instance, while the average carbon footprint for the primary production of PF (high-strength glass fiber, molding) is between 2.42 and 2.67 kg/kg, it ranges from 30.5 to 33.6 for PAI (30% carbon fiber). The latter is also about 14 times more expensive than the former per unit mass.

While the primary production carbon footprint is an indicator of the environmental impact of materials, a life cycle assessment covers other aspects such as the energy consumption for production and storage, transportation-related emissions, and releases of other non-eco materials on curing, amongst others. A full-scale life cycle assessment is beyond the scope of this work. However, a cradle-to-gate life cycle assessment conducted in [84] found that the production of 1 kg of phenol formaldehyde generates 2.88 kg of CO_2_-eq of greenhouse gases. This value is 11.6 percent higher than the primary production CO_2_ reported in Ansys Granta for glass-fiber-reinforced phenol formaldehyde. The study, however, includes other greenhouse gases expressed in CO_2_ equivalent. In addition to this, the ozone depletion potential and human toxicity (cancer effects) of producing 1 kg of phenol formaldehyde are calculated as 2.25 × 10^−11^ kg CFC 11-eq./FU and 9.88 × 10^−9^ CTUh/FU, respectively, while the overall impact of producing 1 kg of phenol formaldehyde using the Eco-99 impact assessment method using a human health impact, ecosystem quality and resource depletion is 2.84 Pt.

While concerns remain over the use of phenol formaldehyde, its use in the building industry is attributed to its low free phenol and formaldehyde contents [85].

The shortlisted materials are promising alternatives that could withstand the impact of the compressive forces arising from the horizontal components of the overburden pressure and the impact of creeping formations over extended periods in the presence of reservoir fluids under HPHT conditions. It is established in the literature that HPHT wells present peculiar challenges to the performance of Portland cement as a well barrier [86]. Given that cement is brittle and knowing the impact of thermally induced stress on cement integrity, its strength deterioration and debonding are accelerated under HTHP conditions [87]. In addition, high-temperature conditions are also known to accelerate the chemical degradation of Portland cement in the presence of aggressive substances such as brine and weak acids, which are often present in reservoirs [19,87,88]. In contrast, an aging study conducted on phenol formaldehyde composite found that the material retained its tensile strength during accelerated aging conducted at 150 °C. Additionally, a time–temperature creep study of the material at 100 °C indicated that after a period of 50 years, the materials would still require a stress level of 89 percent of its ultimate strength to fail [89].

Furthermore, phenol formaldehyde is identified as one of the main thermosetting resins used in the plugging of loss circulation zones during drilling of deep and ultra-deep wells. This application of phenol formaldehyde is driven by a combination of the favorable mechanical properties of the cured material, its corrosion and its wear resistance and thermal stability, which support its performance in HPHT conditions [89,90]. The relatively low price of the material, and the good ablative and flame retardancy properties, is the key reason for its popularity in thermal protection systems. However, its thermal properties have been challenged as insufficient for certain applications in the aircraft industry, where additives are required to improve the thermal performance of phenolic resins [91,92]. In high-temperature oil and gas formations where conventional gellants are not viable, phenol formaldehyde cross-linked gellant has demonstrated viability for applications in water shut-off at 140 degrees centigrade [93]. Another study on the use of the Bakelite phenol formaldehyde in the oil and gas industry used it as an internal coating to improve the corrosion resistance of materials. The success of Bakelite-coated materials is attributed to the chemical resistance of phenolics to CO_2_, oxygen and brine mixtures, hydrochloric and hydrofluoric acids. In these applications, the coatings are stable up to 204 °C [94]. There is a scarcity of similar studies for the other shortlisted materials. Studies on the successful application of phenol formaldehyde in harsh conditions in oil and gas support the robustness of the Granta and TOPSIS-AHP material selection strategy as a tool for material selection for the abandonment of high-pressure high-temperature wells.

In normally pressured wells where the barrier materials are less likely to fracture in compression, the performance of Portland cement is often considered sufficient in the oil and gas industry even in the presence of a CO_2_/brine mixture. In the presence of CO_2_/brine and the absence of a continuous flow pathway, the performance of Portland cement could be enhanced through a self-healing carbonation reaction. Readers may refer to the works of [95,96,97] for greater insight into the impact of carbonation on cement’s performance as a barrier.

The material selection conducted in this work is aimed at finding alternative materials with mechanical and chemical stability to withstand HPHT reservoir conditions. While these are key to material performance, the adoption of the top-ranked alternative materials could be faced by several challenges. The primary challenge to alternative barrier materials in the plug and abandonment of wells is the lack of regulatory support [98] and insufficient support from operators to drive field trials for alternative barriers. In addition to this, the deployment of carbon- and glass-fiber-reinforced materials using conventional pumping technologies could be challenging depending on the fiber length and could require modifications to conventional pumping technology. The fibers could also create a near wellbore bridge that affects the resin injectivity. As a result, it is important to investigate the properties of the non-reinforced polymers and compare them to the properties of Portland cement. For instance, the average compressive strengths of PAI unfilled and PF unfilled are 173 and 92.85 MPa, respectively, from the ANSYS Granta materials selector. In comparison, the average compressive strength of ordinary Portland cement is 19.7 MPa. Class-G Portland cement, which is applicable in HPHT wells, is reported to have a compressive strength of 58 MPa [7]. PAI and PF unfilled are also durable in brine, weak acids and organic solvents according to the Granta database. Solid-free resins also have an injectivity advantage over cement. The long-term performance of PF under reservoir conditions is confirmed in studies conducted by [89,90,93,94], as discussed above. For its part, PAI also has excellent mechanical properties under high temperatures and high resistance to chemical attacks [99,100].

The higher cost of materials, in comparison to Portland cement, is another challenge facing the adoption of alternative materials. However, the cost element of materials becomes less important when viewed from the risk of failure, overall project cost and cost of remediation perspectives. The long-term performance of cement, a relatively cheaper material, has been criticized by various authors, especially as a barrier material in HPHT wells [101].

The availability and ease of sourcing of the selected alternatives may also vary in different countries. A recent attempt to purchase phenol formaldehyde resin in the UK revealed that most suppliers of the material have a specific minimum volume below which they would not accept a supply order. In addition to this, its transportation over long distances require cold conditions. For instance, to achieve a shelf life of over 6 months, the Hexion phenol formaldehyde resin supplied under the trade name CELLOBOND™ J2027X01 by Caleb Technical Limited, Britania Porth, United Kingdom requires a storage temperature of less than 10 degrees centigrade. While this is generally not a major concern in the winter, it has a significant impact on the storage cost and increases the risk of curing at the surface in warmer climates, especially in the situation of an equipment failure during well abandonment operations. Given that thermosets cure exothermically, it is important to quantify the impact of the heat generated during the large volume curing of phenol formaldehyde on the integrity of cement behind the casing in high-temperature wells.

Another challenge to the application of the selected materials is the need for specially trained personnel with a good understanding of polymer behavior to customize the resin solutions for each abandonment scenario based on the individual well conditions. A poor choice of hardener-to-resin ratio could result in premature or late curing downhole. These materials also require additional safety measures, especially in enclosed spaces. The manufacturers of phenol formaldehyde, for instance, recommend their handling in properly ventilated conditions. Well abandonment operations are generally conducted in open-air locations. This generally reduces the health hazards to personnel. However, personal protection equipment should be worn at every point of handling the material to protect the skin, the eyes and the respiratory tract.

Regardless of the above challenges, cement has limitations in complex well scenarios where material injection into the producing reservoir is required during abandonment. In other instances, such as parted tubing, with sustained casing pressures that require section milling, resin solutions have proven to be a cost-effective solution outcompeting cement in the total project cost even in situations where the material cost is 10 times higher than the cost of cement per unit weight. In one instance, a resin solution adopted to seal micro-channels in a sour gas well in China cost only 33 percent of the operator’s projected expenditure in a cement-based solution [102].

In a complex project by Oceaneering for a Gulf of Mexico operator, the adoption of a resin solution delivered a permanent barrier at around 50 percent of the estimated cost of a cement-based solution [103,104].

As shown in Table 11, according to the cost and risk benefit analysis of cement and resin barrier materials, the cost benefit of resin-based solutions does not come from their price per unit volume but from the operational efficiency driven by the reduced milling length in wells that require section milling, the reduced length of the barrier material required to achieve good zonal isolation and the little to zero chance of re-entry for remedial abandonment. In certain wells, the adoption of resin barrier materials also enables the implementation of rigless well abandonment. Note that the values in Table 11 are specific to the referenced projects. The values for phenol formaldehyde are estimated to be fifteen percent more than the average values for resins in the referenced published literature. The rig rates are variable and the milling rates and material volumes also vary based on the casing size and nature. The table is provided as a guide and readers wishing to build on this should take these variations into consideration.

## 5. Comparison of Results with Other Methodologies for Assessing Alternative Barriers

The decision-making framework leading to the selection of well abandonment materials by service contractors and operators is scarcely available in the published literature. However, the costs, case histories and regulatory requirements are common factors that are known to play significant roles in the selection of materials for well abandonment. The combination of these factors limits the adoption of alternatives to cement in well abandonment operations, given the initial cost benefits and long use history of Portland cement in the oil and gas industry.

Another approach to material selection is simulation and modeling based on the well conditions. While this approach considers the long-term performance of materials under downhole conditions, the process of determining which material is modeled could also be constrained to popular material choices. However, the multi-criteria decision-making approach adopted in this work combines regulatory requirements, as well as the cost and performance properties of materials, to explore a broad scope of engineering materials, using critical performance properties to shortlist and rank materials for use in the plug and abandonment of HPHT wells. The top-ranking materials could then be studied further through simulations and confirmatory laboratory tests.

In comparison, the NASA Technology Readiness Level approach to the ranking of well barrier materials adopted in [98] ranks well abandonment barrier materials based on five factors, including the maturity, applicability, risk, cost and benefits. The authors emphasized that the current structuring of regulations around cement limits the potential for the replacement of cement as a primary abandonment material in the short term. The ranking table obtained by the authors shows that the thermosetting polymers and composites have benefit score of 6 against a score of 4.7 for cement. Cement ranks relatively better than thermoset across other metrics adopted by the authors, including the maturation of the technology. The rankings of thermoplastic polymers and composites, as well abandonment materials, are lower than that of cement across all the considered metrics.

In the current ranking of materials for the abandonment of HPHT wells using a combined TOSIS-AHP approach, the choice materials, PF and PAI materials, are thermosetting and thermoplastic materials, respectively, and rank above Portland cement. While this conclusion is at variance with the ranking generated in [98], it is important to highlight that the ranking criteria used in both approaches are a leading cause of the differences. The inclusion of technology, readiness and maturity favors tested materials with a long use history over alternatives. However, both approaches show that thermosetting resins have mechanical properties and long-term degradation resistance benefits over Portland cement. The ranking difference obtained via the two approaches highlights the importance of non-material property factors in the design and development of alternative barrier materials for well abandonment.

## 6. Limitations of the Study and Future Research

While the methodology adopted in this research has been proven to be effective in multi-criteria decision studies, it is important to highlight that the material screening tool, ANSYS Granta, does not provide a detailed description of the solutions in which the durability of materials is tested. There are rather broad classes, such as weak acids, strong acids, etc., but specific acids and their concentrations are not defined for durability studies.

In addition to the above, the aging periods for the material durability tests in solutions used for their performance classification in Ansys Granta are not defined. This could have implications for the field performance of the materials. As a result, further studies on the selected materials in controlled laboratory conditions of known corrosive agents with defined concentrations and aging periods remain an open area for further research.

MCDM has proven effective in material selection for engineering design; however, it is important to note that both TOPSIS and the AHP are subject to order reversal, a phenomenon where the ranking of materials changes with the removal or addition of materials from the choice alternatives. This is also observed with the inclusion or exclusion of material properties in the list of selection criteria, as indicated in the sensitivity analysis. Companies and researchers intending to adopt this approach for material ranking must first determine the critical properties relevant to the design.

This study provides a ranking of materials based on the selected properties, but further experimental work will be needed to qualify these alternatives as acceptable barrier materials for HPHT wells by regulatory bodies.

While the material cost has been included in this study, it is important to note that the actual material costs could vary in the future, and this will have implications for the material ranking. In addition to this, potential improvements in the properties of the ranked materials with new synthesis techniques have not been captured in this study.

## 7. Conclusions

The shortlisted materials are promising alternatives to withstand the impact of the compressive forces arising from the horizontal components of the overburden pressure, the impact of creeping formations and the weight factor of the overlaying barrier material over extended periods in the presence of corrosive reservoir fluids under HPHT conditions.

The compressive strength, fracture toughness, thermal distortion resistance and Poisson’s ratio of the materials are shown to play significant roles in the mechanical performance of well barriers in the HPHT scenario, where forces from the impact of overburden and formation pressures can generate crushing effects and lead to the development of fractures in the barrier materials.

In contrast to the traditional method of choosing well barrier materials based on case histories and experience, an integrated Ansys Granta selector and TOPSIS-AHP multi-criteria decision-making model are applied in this work for the screening and ranking of materials to understand their potential usefulness as permanent barriers in HPHT wells. This approach is scalable to fit a smaller or larger number of selection criteria, varying scenarios of well abandonment and other oil and gas operations that require material selection. In addition, this approach can be applied in material selection studies in the development of new fields where a directly referenceable case history is non-existent.

While Portland and high alumina cement could meet the performance requirements in normally pressured wells, their long-term integrity in HPHT wells is questionable. Additionally, in wells with acidified reservoir fluids, the presence of fractures in Portland cement exposes the well to integrity failures through cement acid interaction.

While cement outperforms the alternatives in this study based on the price per unit volume, operational efficiencies associated with resin solutions and the cost of barrier failures in cement abandoned wells will outweigh this initial advantage. A long-term view of the performance of well barriers is recommended as the basis for P&A material selection, given that abandoned wells are expected, by regulation, to retain their integrity eternally.

## 8. Recommendations for Further Research

Laboratory and simulation studies to assess the long-term behavior of the shortlisted materials are recommended prior to field application as acceptable reservoir barrier materials for the plug and abandonment of HPHT oil and gas wells in aggressive environments. These experiments could include the impact of the time of material exposure to different mixtures and concentrations of reservoir fluids on the final properties and the impact of compression during curing on the material properties.

Alternative synthesis methodologies for the top-ranking materials that will reduce their environmental footprint and cost should be investigated to ease their adoption in plug and abandonment.

The possible use of the shortlisted materials as additives in oil well cement is recommended. In this, the curing mechanism of the cement–resin mixture, the inter-facial bonding between the cement and the material in the cured state, and the other mechanical, chemical and thermal properties of the cured mixture should be examined.

The performance of the non-reinforced resins of the materials ranked in this study should be investigated to determine the range of their usefulness in well abandonment, given that solid-free resins have a flow and penetration advantage over solid-containing materials in well abandonment.

In addition to the quantitative study of their performance, a qualitative study of the acceptability of these materials within the industry is also recommended.

## Figures and Tables

**Table 1:** Limit conditions for the selection of well barrier materials in the ANSYS Granta selector.

**Mechanical and Thermal Properties**	
**Property**	**Limiting Value**
Minimum compressive strength	80 MPa
Minimum service temperature	130 °C
Maximum service temperature	220 °C
**Durability in selected fluids**	
**Material operating environment**	**Performance rating**
Water	Excellent
Brine	Excellent
Weak acids	Excellent
Strong acids	Excellent, Acceptable, Limited Use
Organic solvents	Excellent

**Table 2:** Material selection criteria and assigned weights for the TOPSIS analysis.

Selection Criteria	Assigned Weight	Relevance to Performance of Well Barriers
Compressive strength	0.2	This quantifies the ability of well barrier materials to withstand failure due to downhole stress [57]. In high temperature wells where accelerated degradation is a concern, especially in the presence of corrosive agents [18], the value of the compressive strength of barrier materials and its retention play a significant role in ensuring long-term integrity. The compressive strength of materials has also been shown to have an inverse relationship with the permeability [58].
Fracture toughness	0.15	This measures the resistance of a barrier material to crack/fracture propagation. In HPHT wells, both thermally and mechanically induced stresses could lead to fracture initiation in well barrier materials. A good fracture toughness value is therefore relevant to the long-term integrity of well barriers [59]
Poisson’s ratio	0.05	According to [13] materials with higher Poisson’s ratio values are preferred for improving zonal isolation. This property is most relevant in situations where the load on the lower section of the barrier is sufficient to create a horizontal expansion of the material. In this scenario, the material bonding to the formation or casing is improved.
Thermal distortion resistance, TDR	0.1	This property measures the ability of barrier materials to maintain dimensional stability at elevated temperatures. Thermal distortion of a barrier material can lead to positional instability. This leads to debonding and the creation of migration pathways, which are established causes of integrity failures in abandoned wells [13,60].
Bulk modulus	0.1	The bulk modulus of materials represents their resistance to volumetric change under compressive force; for example, eternal uniform hydrostatic pressure. Its role in autogenous shrinkage in cement paste is discussed in [61]. A weight factor of 0.1 has been assigned to this property to reflect its relevance in well abandonment materials.
Shear modulus	0.1	Sections of the well abandonment materials could be exposed to varied directions of forces with a cumulate shearing effect. The shear modulus of a material is a measure of its ability to resist deformation under a shear force. According to [42], the shear modulus also affects the loading capacity and failure modes of materials. It is established that the shear modulus of materials is affected by the temperature and pressure [62]. Its degradation in cement is also affected by the number of loading cycles, cement content, curing days, cyclic shear strain amplitude and loading frequency [63].
Durability in water, brine, organic and weak acids	0.15	This measures the long-term degradation behavior of well barriers on exposure to wellbore fluids under downhole conditions. Depleted reservoirs contain a mixture of residual hydrocarbon, brine and, in some instances, acids such as carbonic acids, etc. Materials that do not degrade on interaction with well fluids are preferred candidates for permanent abandonment. For a summary of studies on the interaction of various well abandonment materials with reservoir fluids, readers may refer to [9].
Durability in strong acids	0.05	The presence of strong acids is often a result of acid treatment in enhanced oil production [64]. However, evidence from [65,66] suggests that the large volume ratio of brine in depleted reservoirs will have a concentration dilution effect on strong acids in late-life wells. Given that they will not be present in every well, the weight assigned to this material’s durability to strong acids for the sake of this study is 0.05. In specific well conditions, where they are confirmed as present, a higher weight factor will be required in the ranking process.
Average price	0.1	In the cost per unit property material selection method, the cost per unit volume or property carries a major weight in the ranking process [33]. However, in the plug and abandonment of wells, the cost should not be limited to the initial material processing and purchase. The financial, environmental and health consequences of an abandonment material failure should be considered, in addition to potential reputational damage to companies that could arise from hydrocarbon leakages from their decommissioned assets. The price available within the ANSYS Granta software, which has been used in this study, is the price per unit volume.
Sum of weights	1	

**Table 3:** AHP scale for pairwise comparison.

Numerical Scale	Definition	Explanation
1	Equal importance	Two compared criteria contribute equally to the selection objective.
3	Moderate importance	Considered criterion is moderately more important than the one it is being compared to.
5	Strong importance	A criterion is strongly favored over the one it is compared to.
7	Very strong importance	A criterion is strongly favored, and its dominance is demonstrated in practice.
9	Absolute importance	One criterion is affirmed as more important than another in the highest possible order.
2, 4, 6, and 8	Intermediate values	These are compromise values between the values and positions above.

**Table 4:** Random consistency index for the AHP ranking.

K	1	2	3	4	5	6	7	8	9	10
**RI**	0	0	0.58	0.9	1.12	1.24	1.32	1.41	1.45	1.49

**Table 5:** Material rating in the ANSYS Granta selector based on their durability in specific operating conditions.

Rating	Meaning
Unacceptable	Do not use in the unprotected condition
Limited use	Not recommended, although may be suitable for short-term applications
Acceptable	May require additional protection
Excellent	No degradation in material performance expected after long-term exposure

**Table 6:** Property values of the shortlisted materials for the plug and abandonment of HPHT wells. WBOWA—water, brine, organic solvents and weak acids, TDR—thermal distortion resistance, HSGFM—high-strength glass fiber, molding), GMFHRM—glass- and/or mineral-filled, heat-resistant, molding.

Material	Compressive Strength	Fracture Toughness	Poissom’s Ratio	TDR	Bulk Modulus	Shear Modulus	Stability in WBOWA	Stability in Strong Acids	Average Price
PAI (30% carbon fiber)	250	9.1	0.39	0.05895	34.15	8	4	2	61,150
PAI (30% glass fiber)	260	10.555	0.43	0.02285	26.35	3.78	4	2	56,200
PF (GMFHRM)	201.5	2.885	0.3355	0.0136	16.4	6.065	4	2	3215
PF (HSGFM)	352	6.805	0.325	0.01929	16.5	6.77	4	2	3900
PEI (40% glass fiber)	221	10.03	0.3475	0.01975	12.3	4.58	4	4	7805
PEEK (40% glass fiber)	224	9.305	0.344	0.0136	14	4.875	4	2	72,000
PEEK (30% carbon fiber)	233.5	10.265	0.3505	0.0436	18.25	7.01	4	2	88,200
LCP (45% glass fiber)	145	4.555	0.415	0.07815	37.25	6.5	4	3	11,700
LCP unfilled	90	1.93	0.415	0.042	30.75	5.355	4	3	13,750
Cement (high alumina)	35.45	0.4	0.112	0.06135	97.15	15.81	4	4	355
Portland cement	19.7	0.4	0.22	0.04765	24.35	16.75	3.25	1	161.5

**Table 7:** Material ranking for the plug and abandonment of HPHT wells using TOPSIS. Sum of the assigned weights = 1. Compressive strength (CS), fracture toughness (FT), Poisson’s ratio (PR), thermal distortion resistance, (TDR), bulk modulus (BM), shear modulus (SM), durability in water, brine, organic and weak acids (DWBOWA), durability in strong acids (DSA), average price (AP), tensile strength (TS).

Material		Material Properties/Ranking Criteria												
		CS	FT	PR	TDR	BM	SM	DWBOWA	DSA	AP	Weights Sum			
	Weights	0.2	0.15	0.05	0.1	0.1	0.1	0.15	0.05	0.1	1			
PAI (30% carbon fiber)		250	9.10	0.39	0.06	34.15	8.00	4	2	61,150				
PAI (30% Glass fiber)		260	10.56	0.43	0.02	26.35	3.78	4	2	56,200				
PF (glass- and/or mineral-filled, heat-resistant, molding)		201.5	2.89	0.34	0.01	16.40	6.07	4	2	3215				
PF (high-strength glass fiber, molding)		352	6.81	0.33	0.02	16.50	6.77	4	2	3900				
PEI (40% glass fiber)		221	10.03	0.35	0.02	12.30	4.58	4	4	7805				
Pek (40% glass fiber)		224	9.31	0.34	0.01	14.00	4.88	4	2	72,000				
PEK (30%carbon fiber)		233.5	10.27	0.35	0.04	18.25	7.01	4	2	88,200				
LCP (45% glass fiber)		145	4.56	0.42	0.08	37.25	6.50	4	3	11,700				
LCP UNFILLED		90	1.93	0.42	0.04	30.75	5.36	4	3	13,750				
CEMENT (high alumina)		35.45	0.40	0.11	0.06	97.15	15.81	4	4	355				
Portland cement		19.7	0.40	0.22	0.05	24.35	16.75	3.25	1	161.5				
**Normalized Decision Matrix**											dips d*	**dins da**	**D**	**Rank**
PAI (30% carbon fiber)		0.07	0.06	0.02	0.04	0.03	0.03	0.05	0.01	0.04	0.08	0.10	0.55	2
PAI (30% glass fiber)		0.08	0.07	0.02	0.02	0.02	0.01	0.05	0.01	0.04	0.10	0.10	0.51	4
PF (glass- and/or mineral-filled, heat-resistant, molding)		0.06	0.02	0.01	0.01	0.01	0.02	0.05	0.01	0.00	0.11	0.08	0.43	8
PF (high-strength glass fiber, molding)		0.10	0.04	0.01	0.01	0.01	0.02	0.05	0.01	0.00	0.09	0.12	0.58	1
PEI (40% glass fiber)		0.06	0.06	0.02	0.01	0.01	0.02	0.05	0.02	0.01	0.10	0.10	0.52	3
PEk (40% glass fiber)		0.06	0.06	0.01	0.01	0.01	0.02	0.05	0.01	0.05	0.11	0.08	0.43	9
PEK (30%carbon fiber)		0.07	0.06	0.02	0.03	0.01	0.02	0.05	0.01	0.06	0.10	0.09	0.47	7
LCP (45% glass fiber)		0.04	0.03	0.02	0.05	0.03	0.02	0.05	0.02	0.01	0.09	0.09	0.49	6
LCP UNFILLED		0.03	0.01	0.02	0.03	0.02	0.02	0.05	0.02	0.01	0.12	0.07	0.36	11
Cement (high alumina)		0.01	0.00	0.00	0.04	0.08	0.05	0.05	0.02	0.00	0.11	0.11	0.49	5
Portland cement		0.01	0.00	0.01	0.03	0.02	0.06	0.04	0.01	0.00	0.13	0.08	0.38	10
A*		0.10	0.07	0.02	0.05	0.08	0.06	0.05	0.02	0.00				
Ao		0.01	0.00	0.00	0.01	0.01	0.01	0.04	0.01	0.06				

**Table 8:** AHP pairwise comparison of the selection criteria for barrier materials. Sum of the priority vectors = 1; consistency ratio = 0.033. A consistency ratio of less than or equal to 0.1 is required in the AHP.

	Pairwise Comparison of Material Selection Criteria																
Selection Criteria	CS	FT	TDR	BM	SM	PR	AP	Calculated Normalized Values							Priority Vector	Weight	Ratio of Weights and Corresponding Priority Vectors
Average compressive strength	1	2	2	3	3	2	2	0.27	0.40	0.31	0.21	0.23	0.24	0.15	0.26	1.91	7.39
Fracture toughness	1/2	1	2	3	3	2	2	0.14	0.20	0.31	0.21	0.23	0.24	0.15	0.21	1.57	7.47
Thermal distortion resistance	1/2	1/2	1	3	3	2	2	0.14	0.10	0.15	0.21	0.23	0.24	0.15	0.17	1.29	7.42
Bulk modulus	1/3	1/3	1/3	1	1/2	1/2	2	0.09	0.07	0.05	0.07	0.04	0.06	0.15	0.08	0.53	7.01
Shear modulus	1/3	1/3	1/3	2	1	1/2	2	0.09	0.07	0.05	0.14	0.08	0.06	0.15	0.09	0.65	7.16
Poisson’s ratio	1/2	1/2	1/2	2	2	1	2	0.14	0.10	0.08	0.14	0.15	0.12	0.15	0.13	0.91	7.28
Average price	1/2	1/3	1/3	1/2	1/2	1/2	1	0.14	0.07	0.05	0.03	0.04	0.06	0.08	0.07	0.47	7.09
Sum	3.67	5.00	6.50	14.5	13.0	8.50	13.0								1.00	Average	7.26
																Consistency index	0.043
																Consistency ratio	0.033

**Table 9:** TOPSIS-AHP ranking of the material alternatives.

Materials	Material Properties/Ranking Criteria										
	CS	FT	TDR	BM	SM	PR	AP	Sum of PV			
Priority vectors (PV)	0.26	0.22	0.18	0.07	0.09	0.12	0.06	1.00			
PAI (30% carbon fiber)	250.00	9.10	0.06	34.15	8.00	0.39	61,150.00				
PAI (30% glass fiber)	260.00	10.56	0.02	26.35	3.78	0.43	56,200.00				
PF (glass- and/or mineral-filled, heat-resistant, molding)	201.50	2.89	0.01	16.40	6.07	0.34	3215.00				
PF (high-strength glass fiber, molding)	352.00	6.81	0.02	16.50	6.77	0.33	3900.00				
PEI (40% glass fiber)	221.00	10.03	0.02	12.30	4.58	0.35	7805.00				
PEK (40% glass fiber)	224.00	9.31	0.01	14.00	4.88	0.34	72,000.00				
PEK (30%carbon fiber)	233.50	10.27	0.04	18.25	7.01	0.35	88,200.00				
LCP (45% glass fiber)	145.00	4.56	0.08	37.25	6.50	0.42	11,700.00				
LCP UNFILLED	90.00	1.93	0.04	30.75	5.36	0.42	13,750.00				
CEMENT (high alumina)	35.45	0.40	0.06	17.15	15.65	0.22	422.50				
Portland cement	19.70	0.40	0.05	24.35	16.75	0.22	161.50				
	**Normalized Decision Matrix**							dips d*	**dins da**	**D**	**Ranking**
PAI (30% carbon fiber)	0.10	0.08	0.07	0.03	0.02	0.04	0.02	0.06	0.13	0.69	1
PAI (30% glass fiber)	0.10	0.10	0.03	0.02	0.01	0.05	0.02	0.09	0.13	0.60	5
PF (glass- and/or mineral-filled, heat-resistant, molding)	0.08	0.03	0.02	0.01	0.02	0.04	0.00	0.13	0.08	0.39	8
PF (high-strength glass fiber, molding)	0.13	0.06	0.02	0.01	0.02	0.03	0.00	0.09	0.14	0.62	2
PEI (40% glass fiber)	0.08	0.09	0.02	0.01	0.01	0.04	0.00	0.10	0.12	0.55	6
PEK (40% glass fiber)	0.09	0.08	0.02	0.01	0.01	0.04	0.03	0.11	0.11	0.51	7
PEK (30%carbon fiber)	0.09	0.09	0.05	0.02	0.02	0.04	0.04	0.08	0.13	0.62	4
LCP (45% glass fiber)	0.06	0.04	0.10	0.03	0.02	0.04	0.00	0.10	0.11	0.52	3
LCP UNFILLED	0.03	0.02	0.05	0.03	0.02	0.04	0.01	0.14	0.06	0.31	10
Cement (high alumina)	0.01	0.00	0.08	0.02	0.05	0.02	0.00	0.16	0.08	0.33	9
Portland cement	0.01	0.00	0.06	0.02	0.05	0.02	0.00	0.16	0.07	0.30	11
A*	0.13	0.10	0.10	0.03	0.05	0.05	0.00				
Ao	0.01	0.00	0.02	0.01	0.01	0.02	0.04				

**Table 10:** Sensitivity analysis results.

	D Values in Different Scenarios							
Material	CS = 0.264, BM = 0.072	SM Replaced by TS	CS = 0.215, BM = 0.122	CS = 0.165, BM = 0.172	CS = 0.215, AP = 0.108	CS = 0.165, AP = 0.172	CS = 0.215, TS = 0.122	CS = 0.165, TS = 0.172
PAI (30% carbon fiber)	0.6919	0.7188	0.6961	0.7037	0.6494	0.5938	0.7321	0.7500
PAI (30% glass fiber)	0.5980	0.6300	0.5828	0.5682	0.5676	0.5321	0.6303	0.6380
PF (GMFHRM)	0.3935	0.4004	0.3646	0.3315	0.4237	0.4752	0.3779	0.3575
PF (HSGFM)	0.6203	0.6277	0.5741	0.5197	0.6045	0.6062	0.5838	0.5358
PEI (40% glass fiber)	0.5531	0.5805	0.5337	0.5076	0.5693	0.5975	0.5914	0.6094
PEK (40% glass fiber)	0.5129	0.5358	0.4907	0.4636	0.4787	0.4367	0.5392	0.5506
PEK (30%carbon fiber)	0.6152	0.6411	0.6005	0.5794	0.5574	0.4890	0.6554	0.6766
LCP (45% glass fiber)	0.5214	0.5346	0.5525	0.5895	0.5654	0.6175	0.5598	0.5841
LCP UNFILLED	0.3068	0.3248	0.3352	0.3712	0.3760	0.4536	0.3572	0.3947
Cement (high alumina)	0.3336	0.3024	0.3565	0.3763	0.4087	0.4907	0.3150	0.3193
Portland cement	0.2958	0.2522	0.3242	0.3547	0.3790	0.4678	0.2645	0.2698

**Table 11:** Cost and risk benefit analysis of cement and resin barrier materials.

	Milling Rate in 7″ Casing, m/h			0.4877 [102]	Jack Up Rig Cost, USD/day		133,000 [102]
Material	Average Plug length, m	Mill length, m	Milling time, days	Section milling cost, USD 1000	Material volume, barrels	Risk of leakage	Cost of drilling out failed cement plug USD/500 ft
Cement	100 [105]	20	1.71	227.26	Variable	High	113,636.36 [20]
Resins from the literature	40.2 [102,106]	2 [102]	0.17	22.73	40.67 [102,107]. Variable	Low	N/A
Phenol formaldehyde	46.23	2.3	0.20	26.13	46.77. Variable	Pending field deployment as a permanent plug	N/A

## Data Availability

The original contributions presented in this study are included in the article. Further inquiries can be directed to the corresponding author.
